# Circular RNA ABCB10 contributes to laryngeal squamous cell carcinoma (LSCC) progression by modulating the miR-588/CXCR4 axis

**DOI:** 10.18632/aging.203025

**Published:** 2021-05-18

**Authors:** Jin Zhao, Xing-De Li, Ming Wang, Li-Na Song, Mei-Jiao Zhao

**Affiliations:** 1 Department of Oncology, Cangzhou Central Hospital, Cangzhou, Hebei, China; 2 Department of Radiation Oncology, Cangzhou Central Hospital, Cangzhou, Hebei, China

**Keywords:** laryngeal squamous cell carcinoma, progression, circABCB10, miR-588, CXCR4

## Abstract

Laryngeal squamous cell carcinoma (LSCC) is a common head and neck cancer with a high metastasis and poor prognosis. Circular RNAs (circRNAs) are a type of non-coding RNAs (ncRNAs) with regulatory function and broadly participate in cancer development. However, the correlation of circular RNA ABCB10 (circABCB10) with LSCC remains unclear. Here, we were interested in the role of circABCB10 in the modulation of LSCC progression. Our data demonstrated that the depletion of circABCB10 significantly inhibited the proliferation and induced the apoptosis of LSCC cells. Meanwhile, circABCB10 knockdown was able to remarkably reduce the invasion and migration of LSCC cells. Mechanically, circABCB10 served as a sponge for microRNAs-588 (miR-588) and miR-588 could target and down-regulated chemokine receptor 4 (CXCR4) expression in LSCC cells. The overexpression of CXCR4 or miR-588 inhibitor could reverse circABCB10 depletion-attenuated malignant phenotypes of LSCC cells. Functionally, the depletion of circABCB10 alleviated the tumor growth of LSCC cells in the tumorigenicity analysis of nude mice. The CXCR4 expression was decreased while the miR-588 expression was enhanced by circABCB10 depletion *in vivo*. Thus, we concluded that circABCB10 was involved in the malignant progression of LSCC by regulating miR-588/CXCR4 axis. Our finding provides new insights into the mechanism of circRHOT1 contributing to the development of LSCC. CircABCB10 and miR-588 may be used as potential targets for the treatment of LSCC.

## INTRODUCTION

Laryngeal squamous cell carcinoma (LSCC) is a prevalent and severe malignancy [1, 2]. Despite the improvement of current therapies, such as radiotherapy, chemotherapy, and surgery, the survival rate for advanced LSCC patients remains unsatisfactory [3, 4]. Surgery and radiotherapy are broadly used, and chemotherapy is applied in several curative approaches [3, 4]. The high rate of LSCC recurrence is usually caused by chemotherapy resistance and high invasiveness [5, 6]. Consequently, it is required for the profoundly comprehend the mechanisms of LSCC progression and carcinogenesis for the development of more efficient therapeutic strategies.

Circular RNAs (circRNAs) are the non-coding RNAs (ncRNAs) with crucial roles in the progression of various cancers [7–9]. Increasing evidence has shown that circRNAs are closely correlated with malignant phenotypes of LSCC. It has been reported that circPARD3 facilitates chemoresistance of LSCC cells during cancer progression by suppressing autophagy *via* PRKCI/Akt/mTOR signaling [10]. CircRASSF2 enhances LSCC development by modulating miR-302b-3p/IGF-1R axis [11]. Meanwhile, circular RNA ABCB10 (circABCB10) contributes to the tumorigenesis of various cancer models [12–14], but functions of circABCB10 in LSCC is still unreported.

Another well-recognized small ncRNAs called microRNAs with about 22 nucleotides target 3′ untranslated region (UTR) of various genes to inhibit their expression in many critical biological processes [15–17]. Multiple miRNAs have been identified in the modulation of LSCC. For instance, miR-613 inhibits the progression of LSCC by targeting PDK1 [18]. MiR-154 reduces the LSCC growth by inhibiting GALNT7 [19]. Meanwhile, it has been demonstrated that miR-588 serves as a tumor inhibitor in various cancers, such as lung and breast cancer [20, 21]. Moreover, chemokine receptor 4 (CXCR4) is abnormally expressed in LSCC and contributes to LSCC malignant progression [22, 23]. However, the influence of circABCB10 and miR-588 on CXCR4 in LSCC development remains obscure.

We tried to explore the roles of circABCB10 in LSCC and we reported the new functions of circABCB10 in promoting malignant progression of LSCC *via* the miR-588/CXCR4 axis.

## RESULTS

### CircABCB10 promotes proliferation and inhibits apoptosis of LSCC cells

**Figure 1:**
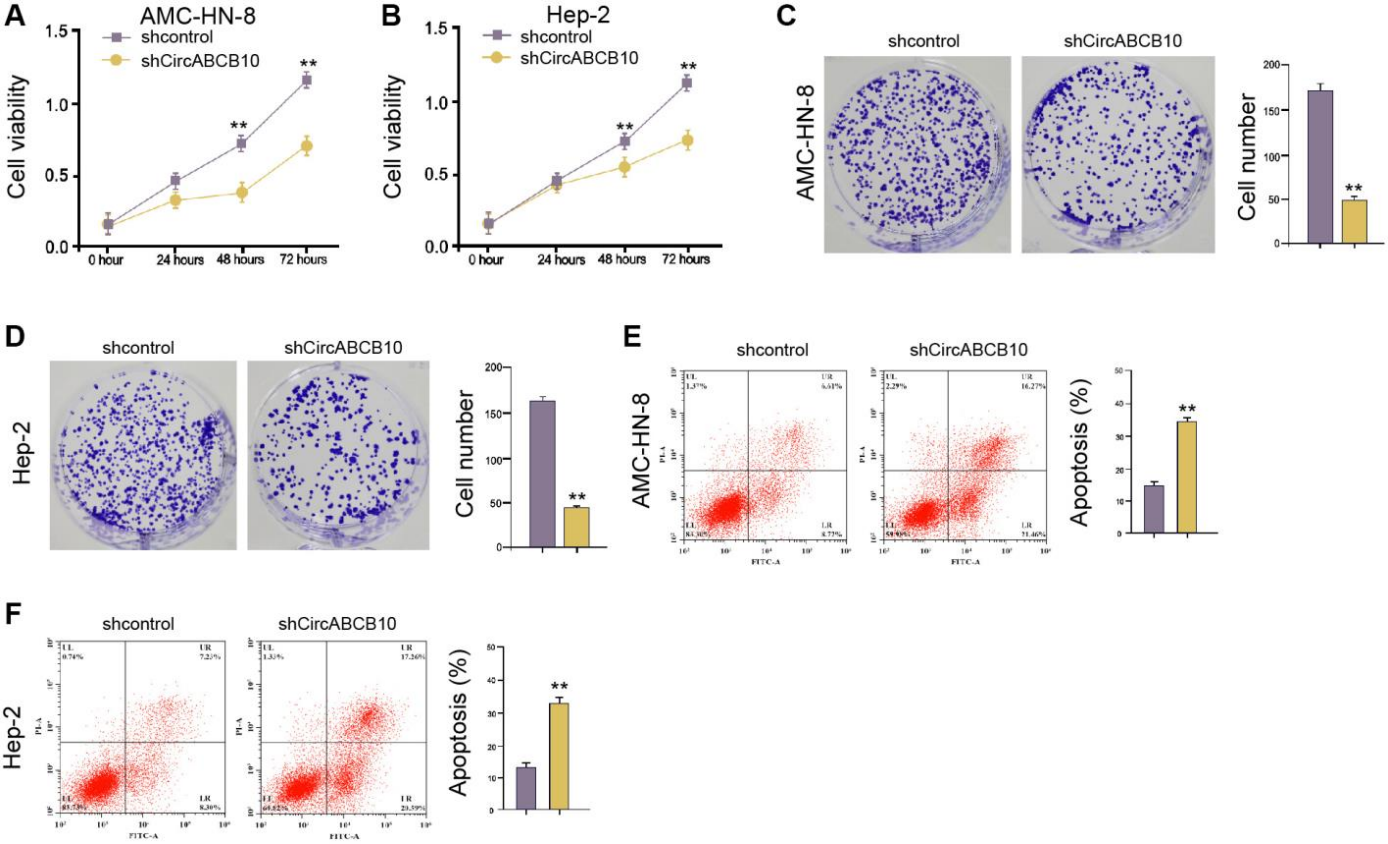
**CircABCB10 promotes proliferation and inhibits apoptosis of LSCC cells.** (**A**–**E**) The AMC-HN-8 and Hep-2 cells were treated with control shRNA or circABCB10 shRNA. (A and B) The cell viability was tested by the MTT assays in the cells. (C and D) The cell proliferation was measured by the colony formation assays in the cells. (E and **F**) The cell apoptosis was analyzed by flow cytometry analysis in the cells. Data are presented as mean ± SD. Statistic significant differences were indicated: ^**^P < 0.01.

We evaluated the effect of circABCB10 on LSCC cell proliferation. MTT assays demonstrated that the circABCB10 depletion repressed cell viabilities (Figure 1A and 1B). Consistently, the colony numbers were suppressed by circABCB10 knockdown (Figure 1C and 1D). Furthermore, cell apoptosis was promoted by the circABCB10 depletion in the cells (Figure 1E and 1F), suggesting that circABCB10 increases proliferation and represses apoptosis of LSCC cells.

### CircABCB10 enhances invasion/migration ability of LSCC cells

**Figure 2:**
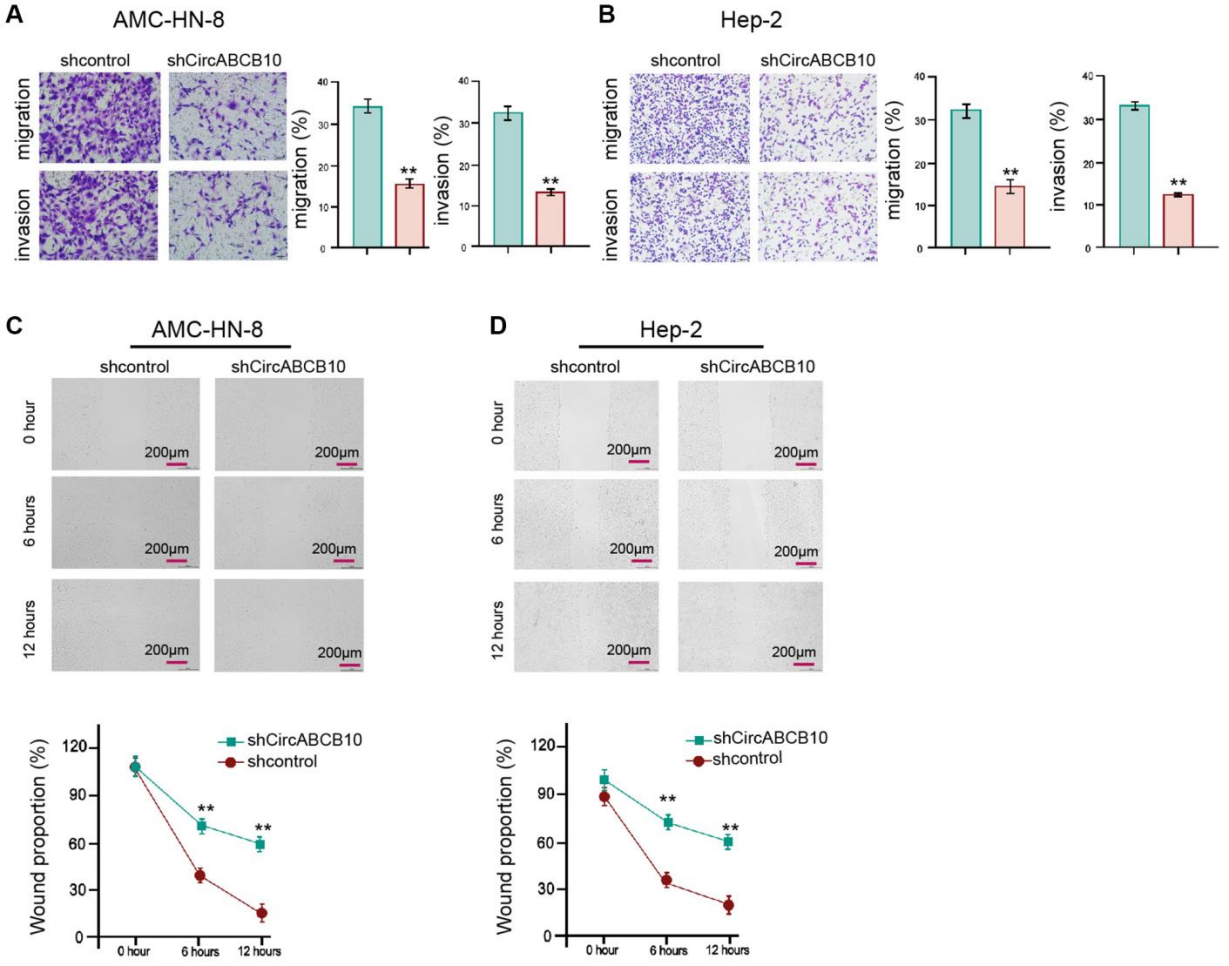
**CircABCB10 enhances invasion and migration of LSCC cells.** [5] The AMC-HN-8 and Hep-2 cells were treated with control shRNA or circABCB10 shRNA. (**A** and **B**) The cell migration and invasion were determined by transwell assays in the cells. (**C** and **D**) The migration and invasion were examined by wound healing assays in the cells. The wound healing proportion was shown. Data are presented as mean ± SD. Statistic significant differences were indicated: ^**^P < 0.01.

We then investigated functions of circABCB10 in modulation of migration/invasion abilities. Transwell assays showed that the capability of migration/invasion was suppressed by depletion of circABCB10 (Figure 2A and 2B). The knockdown of circABCB10 significantly increased wound proportion (Figure 2C and 2D), indicating that circABCB10 is able to induce the migration/invasion of LSCC cells.

### CircABCB10 is able to sponge miR-588 in LSCC cells

**Figure 3:**
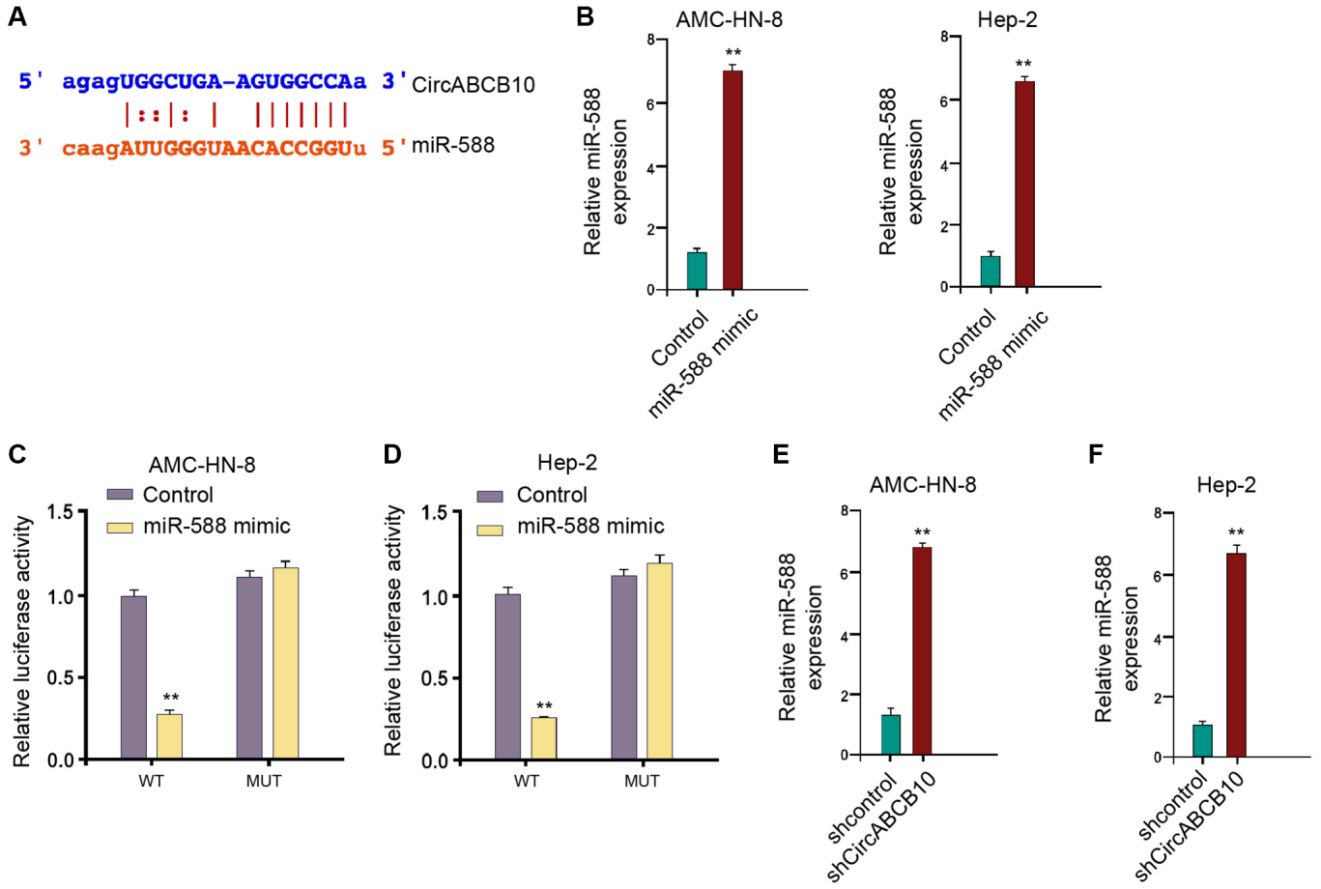
**CircABCB10 is able to sponge miR-588 in LSCC cells.** (**A**) The potential interaction between circABCB10 and miR-588 was identified by the bioinformatic analysis using ENCORI (http://starbase.sysu.edu.cn/index.php). (**B**–**D**) The AMC-HN-8 and Hep-2 cells were treated with the miR-588 mimic or control mimic. (B) The expression levels of miR-588 were measured by qPCR in the cells. (C) The luciferase activities of wild type circABCB10 (WT) and circABCB10 with the miR-588-binding site mutant (MUT) were determined by luciferase reporter gene assays in the cells. (**E** and **F**) The AMC-HN-8 and Hep-2 cells were treated with control shRNA or circABCB10 shRNA. The expression of miR-588 was analyzed by qPCR in the cells. Data are presented as mean ± SD. Statistic significant differences were indicated: ^**^P < 0.01.

Next, we further investigated the mechanism underlying circABCB10-regulated LSCC progression. We found the potential interaction between circABCB10 and miR-588 using a bioinformatic analysis (Figure 3A). The AMC-HN-8 and Hep-2 cells were treated with miR-588 mimic and presented enhanced miR-588 expression (Figure 3B). The miR-588 significantly inhibited luciferase activity of wild type circABCB10, but not circABCB10 mutant (Figure 3C and 3D). Consistently, the depletion of circABCB10 remarkably promoted miR-588 expression in the AMC-HN-8 and Hep-2 cells (Figure 3E and 3F), suggesting that circABCB10 is able to sponge miR-588 in LSCC cells.

### MiR-588 is able to target CXCR4 in LSCC cells

**Figure 4:**
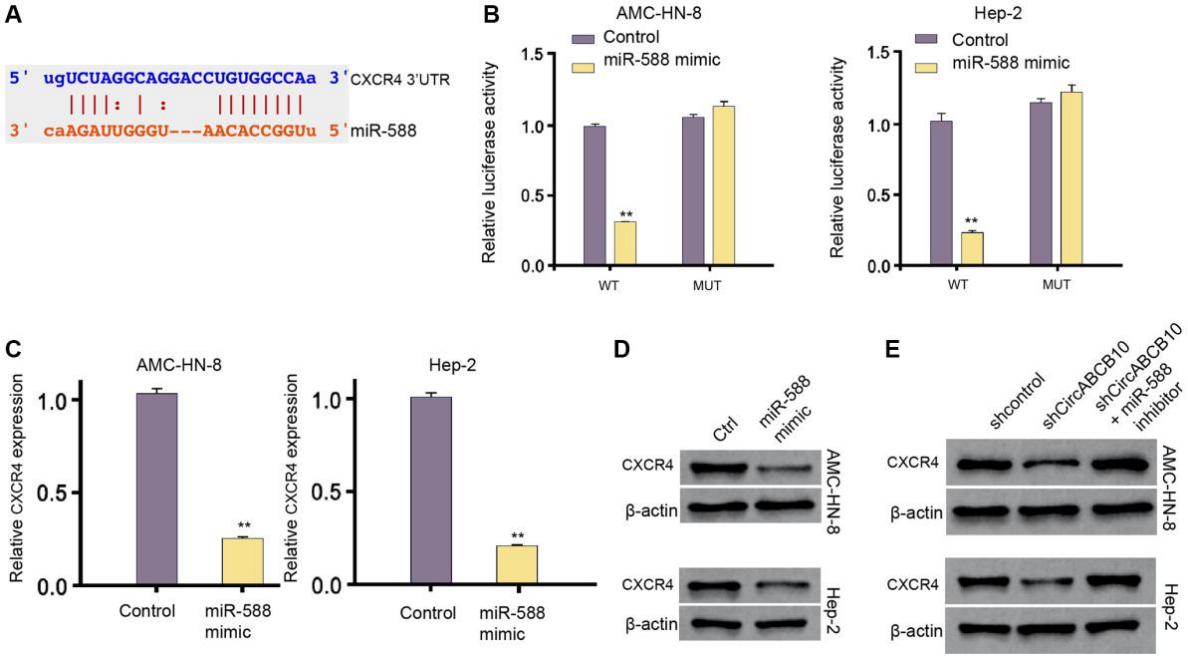
**MiR-588 is able to target CXCR4 in LSCC cells.** (**A**) The interaction of miR-588 and CXCR4 3′UTR was identified by bioinformatic analysis using Targetscan (http://www.targetscan.org/vert_72/). (**B**–**D**) The AMC-HN-8 and Hep-2 cells were treated with the miR-588 mimic or control mimic. (**B**) The luciferase activities of wild type CXCR4 (WT) and CXCR4 with the miR-588-binding site mutant (MUT) were determined by luciferase reporter gene assays in the cell. (**C**) The mRNA expression of CXCR4 was analyzed by qPCR in the cells. (**D**) The protein expression of CXCR4 and β-actin was tested by Western blot analysis in the cells. (**E**) The AMC-HN-8 and Hep-2 cells were treated control shRNA, circABCB10 shRNA, or co-treated with circABCB10 shRNA and miR-588 inhibitor. The protein expression of CXCR4 and β-actin was assessed by Western blot analysis in the cells. Data are presented as mean ± SD. Statistic significant differences were indicated: ^**^P < 0.01.

Next, we also revealed the potential miR-588-binding site within CXCR4 3′UTR in a bioinformatic analysis (Figure 4A). The miR-588 mimic inhibited the luciferase activities of wild type CXCR4, but not CXCR4 mutant (Figure 4B). In addition, the levels of CXCR4 were remarkably down-regulated by miR-588 mimic in the cells (Figure 4C and 4D). Moreover, depletion of circABCB10 significantly reduced expression of CXCR4, and miR-588 inhibitor reversed the result (Figure 4E).

### CircABCB10 contributes to LSCC progression by miR-588/CXCR4 axis

**Figure 5:**
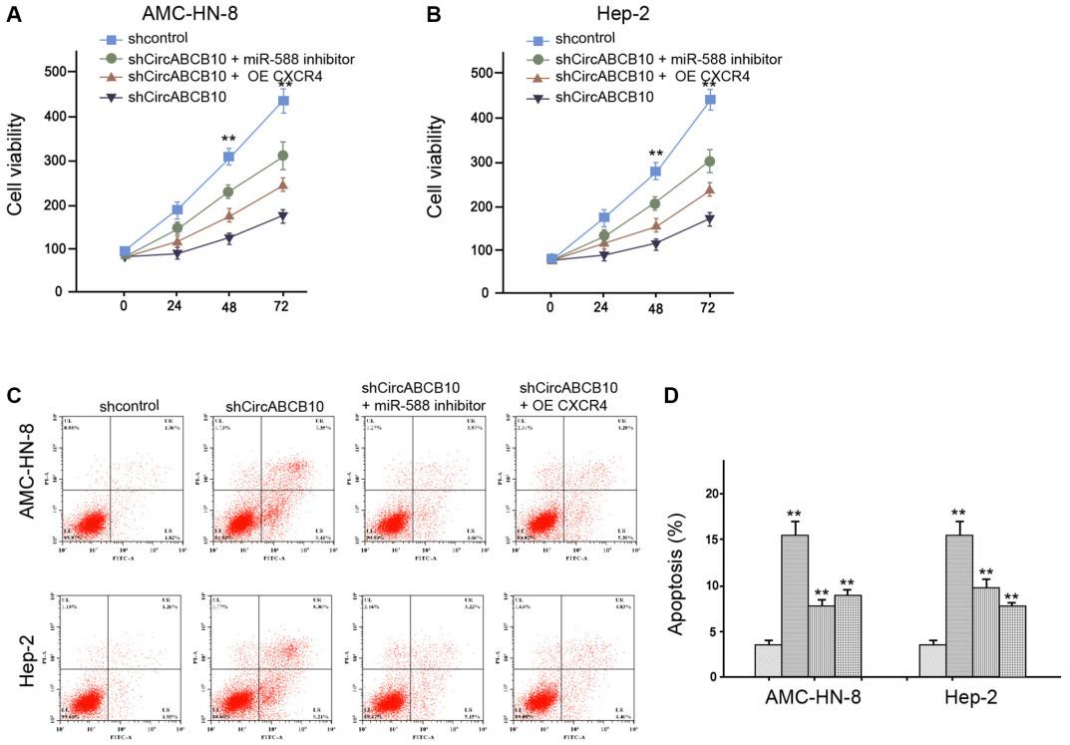
**CircABCB10 contributes to LSCC progression by miR-588/CXCR4 axis.** (**A**–**D**) The AMC-HN-8 and Hep-2 cells were treated control shRNA, circABCB10 shRNA, or co-treated with circABCB10 shRNA and miR-588 inhibitor or pcDNA-CXCR4. (**A** and **B**) The cell viability was analyzed by MTT assays in the cells. (**C** and **D**) The cell apoptosis was tested by flow cytometry analysis in the cells. Data are presented as mean ± SD. Statistic significant differences were indicated: ^**^P < 0.01.

Next, we analyzed the function of circABCB10/miR-588/CXCR4 axis in the regulation of LSCC malignant progression. As expected, the CXCR4 overexpression or miR-588 inhibitor could increase the circABCB10 depletion-inhibited cell viability in the AMC-HN-8 and Hep-2 cells (Figure 5A and 5B). Furthermore, apoptosis was increased by depletion of circABCB10, in which overexpression of CXCR4 or miR-588 inhibitor could reverse this phenotype in the AMC-HN-8 and Hep-2 cells (Figure 5C and 5D). Together these data indicate that circABCB10 enhances LSCC malignant progression by regulating miR-588/CXCR4 axis.

### CircABCB10 promotes the cell proliferation of LSCC *in vivo*

**Figure 6:**
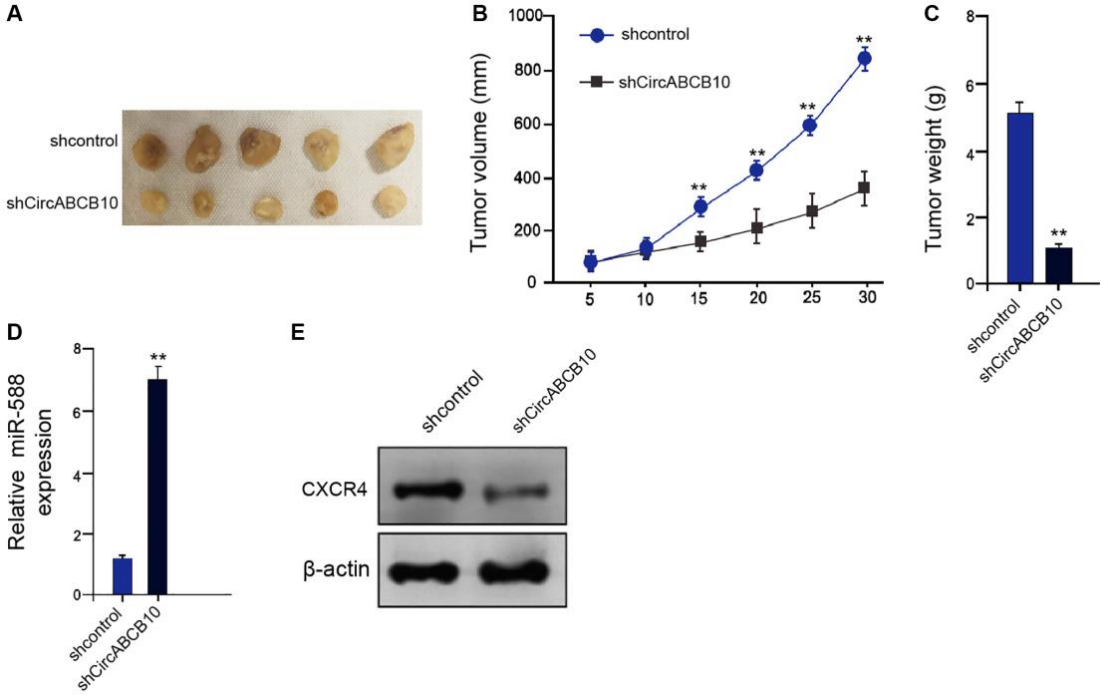
**CircABCB10 promotes the tumor growth of LSCC *in vivo*.** (**A**–**E**) The effect of circABCB10 on tumor growth of LSCC cells *in vivo* was analyzed by nude mice tumorigenicity assay by injected with the AMC-HN-8 cells treated with control shRNA or circABCB10 shRNA. (**A**) Representative images of dissected tumors from nude mice were presented. (**B**) The average tumor volume was calculated and shown. (**C**) The average tumor weight was calculated and shown. (**D**) The expression levels of miR-588 were measured by qPCR in the tumor tissues of the mice. (**E**) The protein expression of CXCR4 and β-actin was assessed by Western blot analysis in the tumor tissues of the mice. *N* = 5. Data are presented as mean ± SD. Statistic significant differences were indicated: ^**^P < 0.01.

Next, the function of circABCB10 in regulating LSCC cell growth was explored *in vivo*. Tumorigenicity analysis showed that the circABCB10 depletion attenuated the tumor growth, as presented by the decreased tumor size (Figure 6A), repressed tumor volume (Figure 6B), and reduced tumor weight (Figure 6C). As expected, the miR-588 expression was up-regulated and CXCR4 expression was down-regulated by circABCB10 knockdown in the tumor tissues of the mice (Figure 6D and 6E). Taken together, these data suggest that circABCB10 promotes the tumor growth of LSCC *in vivo.*

## DISCUSSION

LSCC is the prevailing head and neck cancer with high mortality and widely affects modern people [2]. CircRNAs exert important roles in development of LSCC (Liang, 2017 #5). Nevertheless, the effect of circABCB10 on the tumorigenesis of LSCC is still poorly investigated. We firstly reported that circABCB10 promoted malignant progression of LSCC by the miR-588/CXCR4 axis.

Many previous investigations have shown several circRNAs in the LSCC pathogenesis. It has been reported that circMYLK promotes LSCC progression by miRNA-195/cyclin D1 signaling [24]. The up-regulation of circRNA100290 enhances the LSCC development [25]. Circ103862 contributes to the invasion of LSCC [26]. Besides, circABCB10 enhances migration and proliferation of lung cancer by miR-1252/FOXR2 signaling [27]. CircABCB10 increases the progression of non-small cell lung cancer [28]. CircABCB10 contributes to invasion and proliferation of esophageal squamous cell carcinoma cells by targeting miRNA-670-3p [29]. We demonstrated that circABCB10 enhanced LSCC progression. CircABCB10 promoted the cell growth of LSCC *in vivo*. It demonstrates the critical roles of circABCB10 in the LSCC, indicating informative evidence of the function of circRNAs in modulating LSCC.

As the interacted non-coding regulators of circRNAs, miRNAs are also well-described in LSCC development. MiRNA-26a represses tumorigenesis and proliferation of LSCC by inhibiting CKS2 [30]. CircFLNA elevation promotes LSCC migration [31]. MiRNA-195 suppresses cell invasion, migration, and proliferation in LSCC by down-regulating ROCK1 [32]. MiR-588 as a tumor suppressor is involved in long non-coding RNA GSEC-mediated osteosarcoma progression [33]. Furthermore, it has been reported that CXCR4 contributes to LSCC metastasis by regulating MMP-13/ERK1/2/AP-1 signaling [22]. CXCR4 induces angiogenesis of LSCC [34]. Our mechanical investigation revealed that circABCB10 could serve as a sponge for miR-588 and miR-588 was able to target CXCR4 in LSCC cells. The overexpression of CXCR4 or miR-588 inhibitor could reverse circABCB10 knockdown-inhibited LSCC malignant phenotypes *in vitro.* These data reveal an unreported association of circABCB10 with miR-588 and CXCR4, elucidating a new mechanism involving circABCB10, miR-588, and CXCR4 in LSCC pathogenesis.

In this study, we identified the new function of circular RNA circABCB10 in the development of LSCC, and we found that miR-588/CXCR4 axis was involved in circABCB10-meidiated LSCC progression. Despite the innovation of this study, there are still some limitations of this study. The significance and the relationship of circABCB10, miR-588, and CXCR4 in the clinical LSCC samples were elusive, which deserve to be investigated. Meanwhile, the miR-588/CXCR4 axis might be just one of the downstream factors of circABCB10 in regulating LSCC progression. Other potential mechanisms need to be explored in the future study.

Moreover, we identified that circABCB10 contributed to the LSCC cell invasion, migration, proliferation, and repressed LSCC cell apoptosis *in vitro*. CircABCB10 was also involved in growth of LSCC cells *in vivo*. It implies that targeting circABCB10 may be a potential therapeutic strategy in the clinic, and more evidence needs to be found to validate its therapeutic value. Meanwhile, the expression of circABCB10 in the clinical LSCC samples and the correlation of circABCB10 with the survival rate of LSCC patients needed to analyze to evaluate the diagnostic and prognostic significance of circABCB10.

In conclusion, we discovered that circABCB10 was involved in the malignant progression of LSCC by regulating the miR-588/CXCR4 axis. CircABCB10 and miR-588 may serve as targets for LSCC therapy.

## MATERIALS AND METHODS

### Cell culture

AMC-HN-8 and Hep-2 cells were maintained in the lab and incubated at 37°C and 5% CO_2_ in DMEM (GE, USA) containing FBS (15%, Gibco, USA), 0.1 mg/mL streptomycin (Solarbio, China) and 100 units/mL penicillin (Solarbio, China). The lentiviral plasmids containing circABCB10 shRNA, the pcDNA3.1-CXCR4 overexpression vector, miR-588 mimic and inhibitor were obtained (GenePharma, China) (GenScript, China).

### MTT assays

For MTT assay, AMC-HN-8 and Hep-2 cells were placed in each well of a 96-well plate, followed by the treatment. After 12 hours incubation, 10 μL MTT solution was added in the medium to incubate for another 4 hours, followed by replacement with 150 μL DMSO. The absorbance at 570 nm was detected by a microplate reader.

### Colony formation assays

For colony formation assay, 1 × 10^3^ AMC-HN-8 and Hep-2 cells were suspended as single cell and seeded into a 6-well plate with 1000 cells per well, and incubated for 2 weeks to form visible colonies. The colonies were stained by 1% crystal violate resolved in methanol for 30 minutes, and photographed.

### Transwell assays

The migration and invasion ability of AMC-HN-8 and Hep-2 cells were determined via using a transwell chamber (Corning, USA). To detect migration, AMC-HN-8 and Hep-2 cells (1 × 10^5^ cells/well) were seeded into the upper chambers with FBS-free medium, while the lower chambers were filled with complete DMEM medium. After 24 hours incubation, the membranes of upper chambers were fixed by 4% paraformaldehyde for 15 min, and stained by 0.5% crystal violet for 30 minutes. For cell invasion, the process was similar with that of migration experiment, only that the upper chambers were coated with Matrigel (BD Bioscience, USA).

### Wound healing assay

Wound healing experiment was also performed to detect cell migration. In short, cells were planted in a 6-well plate at a density of 3 × 10^5^ cells per well, and incubated overnight to form a monolayer confluence. Then the monolayer was scratched by a 200 μL pipette tip, and washed with PBS to remove debris. FBS-free medium was added to the wells and cells were cultured for 12 hours. The width of wound was captured and measured at 0, 6, and 12 hours under a microscope.

### Analysis of cell apoptosis

To determine apoptotic cells, we adopted an Annexin V FITC Apoptosis Kit (CST, USA). In short, 2 × 10^5^ AMC-HN-8 and Hep-2 cells were placed in each well of a 6-well plate, digested and suspended in binding buffer. Subsequently, the cells were stained by Annexin-V for 20 minutes at room temperature, followed by staining with PI. The samples were analyzed on a C6 flow cytometer (BD Biosciences, USA).

### Luciferase reporter gene assay

The sequence of circABCB10 and 3′UTR region of CXCR4 were cloned into the pmiR-Glo vector (Promega, USA). Cells were transfected with the WT or Mut along with d miR-588mimics or NC for 24 hours. The cells were lysed and the luciferase activity was measured by a dual luciferase detection kit (Promega, USA).

### Quantitative reverse transcription-PCR (qRT-PCR)

The cells were lysed by a Trizol reagent (Invitrogen, USA). The cDNA was obtained from total RNA (TaKaRa, China) and quantified by SYBR-Green (Takara, China). The primer sequences: circABCB10 F: 5′-CTAAGGAGTCACAGGAAGACATC-3′, R: 5′-GTAGAATCTCTCAGACTCAAGGTTG-3′; miR-588: 5′-CCGCTATTGCACATTACTAAGTTGCA-3′; CXCR4 F: 5′-TTGTTTCGCAAGCTTCCGTT-3′, R: 5′-ACGTGGGCATTTGTCACGAT-3′; GAPDH F: 5′-AACGGATTTGGTCGTATTGGG-3′, R: 5′-CCTGGAAGATGGTGATGGGAT-3′.

### Western blot analysis

Total protein was extracted from AMC-HN-8 and Hep-2 cells using a RIPA lysis reagent (Sigma, USA), quantified by using a BCA kit (Sigma, USA), separated in the SDS-PAGE gel, and transferred to PVDF membranes. Subsequently, the blots were soaked in blocking buffer for 15 minutes, and incubated in diluted primary antibodies against CXCR4 (Abcam, USA) and β-actin (Abcam, USA), overnight at 4°C. Next day, the blots were washed with PBS and incubated with HRP-conjugated secondary ani-mouse or anti-rabbit antibody, accordingly. All antibodies used in this work was purchased from Abcam (USA) and diluted as instructions of the manufacturer. The blots were visualized by using the ECL solution (Beyyotime, China) and captured in a gel imaging system (BioRad, USA).

### Analysis of tumorigenicity in nude mice

AMC-HN-8 cells (1 × 10^7^, 100 μL) were subcutaneously injected into the left fat pad of Balb/c nude mice (*n* = 5) aged 4-week. Five days after inoculation, the mice were sacrificed and tumors were scaled. The tumor size (volume = 0.5 × width^2^ × length) and body weight were measured at the indicated time points. Animal care and method procedure were authorized by the Animal Ethics Committee of Cangzhou Central Hospital, was carried out in accordance with the National Institutes of Health guide for the care and use of Laboratory animals (NIH Publications No. 8023, revised 1978).

### Statistical analysis

Each experiment was repeated three times and the data was presented as means ± SD. Statistical analysis was carried out by using a Graphpad prism. Mann-Whitney U and one-way ANOVA were adopted to evaluate differences between two or more groups. *P* < 0.05 was set as threshold for statical significance.
